# Fecal Protease Activity Is Associated with Compositional Alterations in the Intestinal Microbiota

**DOI:** 10.1371/journal.pone.0078017

**Published:** 2013-10-17

**Authors:** Ian M. Carroll, Tamar Ringel-Kulka, Laurent Ferrier, Michael C. Wu, Jennica P. Siddle, Lionel Bueno, Yehuda Ringel

**Affiliations:** 1 Division of Gastroenterology and Hepatology, Department of Medicine, the University of North Carolina at Chapel Hill, Chapel Hill, North Carolina, United States of America; 2 Gillings School of Global Public Health, the University of North Carolina at Chapel Hill, Chapel Hill, North Carolina, United States of America; 3 Department of Biostatistics, Gillings School of Global Public Health, the University of North Carolina, Chapel Hill, Chapel Hill, North Carolina, United States of America; 4 INRA, UMR1331 Toxalim, Neuro-Gastroenterology & Nutrition group, Toulouse, France; Institut Pasteur de Lille, France

## Abstract

**Objective:**

Intestinal proteases carry out a variety of functions in the gastrointestinal (GI) tract. Studies have reported that elevated enteric proteases in patients with GI disease can alter intestinal physiology, however the origin (human vs. microbial) of elevated proteases in patients with GI disease is unclear.

**Aim:**

The aim of this study was to investigate the association between protease activity and the microbiota in human fecal samples.

**Design:**

In order to capture a wide range of fecal protease (FP) activity stool samples were collected from 30 IBS patients and 24 healthy controls. The intestinal microbiota was characterized using 454 high throughput pyro-sequencing of the 16S rRNA gene. The composition and diversity of microbial communities were determined and compared using the Quantitative Insights Into Microbial Ecology (QIIME) pipeline. FP activity levels were determined using an ELISA-based method. FP activity was ranked and top and bottom quartiles (*n*=13 per quartile) were identified as having high and low FP activity, respectively.

**Results:**

The overall diversity of the intestinal microbiota displayed significant clustering separation (*p* = 0.001) between samples with high vs. low FP activity. The *Lactobacillales, Lachnospiraceae*, and *Streptococcaceae* groups were positively associated with FP activity across the entire study population, whilst the *Ruminococcaceae* family and an unclassified *Coriobacteriales* family were negatively associated with FP activity.

**Conclusions:**

These data demonstrate significant associations between specific intestinal bacterial groups and fecal protease activity and provide a basis for further causative studies investigating the role of enteric microbes and GI diseases.

## Introduction

Proteases, or proteolytic enzymes, catalyze the breakdown of proteins by hydrolysis of peptide bonds. Compared to all other organs in the human body, the gastrointestinal (GI) tract contains the highest levels of endogenous and exogenous proteases [1]. Initially the function of proteases was considered to be the breakdown of protein relevant to food digestion and intracellular protein turnover; however it was discovered that precise cleavage of proteins by proteases leads to a very subtle means of regulation [2]. It is now known that proteases are involved in diverse processes such as cell-cycle progression, cell proliferation and cell death, DNA replication, tissue remodeling, coagulation, wound healing and the immune response [3]. Indeed, proteolytic activity is tightly regulated to prevent any destructive activity of proteases.

Protease-related genes make up approximately 2% of the mammalian genome and host proteases significantly contribute to the enzymatic content of the GI tract. However, the enteric microbiota is also a substantial source of serine, cysteine, and matrix metalloproteinases (MMPs) in the intestine [4-6]. This is exemplified by the reduction of colonic bacteria densities and protease activity by oral administration of antibiotics to mice [7]. Additionally, bacterial proteolytic activity in the intestine is reported to be ubiquitous and independent of inflammation [8]. 

Several studies have reported elevated levels of fecal protease activity in patients with certain GI diseases including inflammatory bowel diseases (IBD, e.g., ulcerative colitis) and irritable bowel syndrome (IBS) [6,9-14]. However, the origin of fecal proteolytic activity, host or microbial, was not determined in these studies. In addition, to date there are limited data regarding which specific intestinal bacterial groups are associated with enteric protease activity. Our study investigates the hypothesis that intestinal protease activity in humans correlates with specific enteric bacterial taxa. Thus, we carried out high throughput sequencing of the 16S rRNA gene to characterize the microbiota in fecal samples with a range of protease activity. Given the reported increase in protease activity in irritable bowel syndrome (IBS) patients (refs), we performed our analysis on fecal samples from healthy individuals and patients with various severities of IBS symptoms to enable the capture of a wide range of fecal protease activity.

## Materials and Methods

### Ethics Statement

The study was approved by the UNC Internal Review Board (IRB) and all subjects provided written consent prior to participation in the study.

### Sample Collection and Preparation

Fecal samples were collected from 54 subjects (30 patients with IBS and 24 healthy controls). All subjects were 18 years or older, and of any gender, race, or ethnicity. Healthy controls had no recurring GI symptoms. Patients had active GI symptoms and met the Rome III criteria for IBS. Participants were excluded if they had a history of treatment with antibiotics, anti-inflammatory agents, or if they had intentionally consumed probiotics two months prior to the study. An eight-week wash-out period was required for subjects who reported intentional consumption of antibiotics or probiotics prior to enrollment. All subjects were recruited from the Chapel Hill general population and from the University of North Carolina (UNC) healthcare outpatient clinics. The study was approved by the UNC Internal Review Board (IRB) and all subjects provided written consent prior to participation in the study. 

Fresh stool samples were collected from all 54 subjects on site when possible during a single study visit at UNC as previously described [15]. Subjects unable to provide stool samples at the visit were instructed to collect a specimen at home and return it to study staff at the same morning. Following delivery, fecal samples were placed in a cooler (4°C) until it reached the laboratory. At the laboratory each sample was homogenized, divided into aliquots and stored at -80°C for future DNA isolation and molecular microbiological analysis. It has previously been shown that the composition of the microbiota [16] and protease activity [17] is stable in fecal samples maintained at 4°C.

### Detection of Fecal Protease Activity

FP activity was quantified in all stool samples using an ELISA-based method as previously described [6,10]. In brief, stool samples were thawed at 4°C. 1 g of each stool sample was transferred to 4 mL of reaction buffer (0.15 mol/L NaCl and 20 mmol/L Tris-HCl) and homogenized. Coarse particles were removed from fecal homogenates by filtration with a 0.8-μm size syringe filter (Nalgene; Nalge, New York, NY) after centrifugation at 4500 rpm for 10 minutes at 4°C. 25 μL of supernatants from fecal homogenates were incubated with 1 mL of reaction buffer and 1 mL of 0.5% (w/v) azocasein (Sigma, St.-Quentin, France) at 40°C for 20 minutes. The reaction was stopped by adding 1 mL of 10% (v/v) trichloroacetic acid (Sigma). After centrifugation at 4500 rpm for 10 minutes at 4°C, absorption of the clear supernatant was measured at 366 nm and compared with standard curves obtained from a titration series of azocasein. Protein concentration of the filtered supernatant of colonic content was assessed with the BCA-200 Protein Assay Kit (Pierce, Rockford, IL), with bovine serum albumin as a standard. Protease activity was expressed as units of trypsin activity per mg of protein. Two different protease inhibitors (aprotinin and soybean tryptase inhibitor) were used to confirm assay specificity.

### Isolation of fecal DNA

Bacterial DNA was isolated from a total of 54 fecal samples using a phenol/chloroform extraction method combined with physical disruption of bacterial cells and a DNA clean-up kit (Qiagen DNeasy® Blood and Tissue extraction kit [Qiagen, Valencia, CA]) as previously described [15].

### 454 pyro-sequencing of 16S rRNA genes

Bacterial community composition in isolated DNA samples was characterized by amplification of the V1-3 (forward, 8f: 5'-AGAGTTTGATCMTGGCTCAG-3'; reverse 518r: 5'-ATTACCGCGGCTGCTGG-3') variable regions of the 16S rRNA gene by polymerase chain reaction (PCR) as previously described [15]. These regions were chosen as it has been reported that multiple regions provide better taxonomic resolution [18]. 

### Analysis of 16S rRNA sequences using the QIIME pipeline

16S rRNA sequence data generated by the 454 GS FLX Titanium sequencer was processed by the quantitative insights into microbial ecology (QIIME) pipeline[19]. Briefly, sequences that were less than 200 bp or greater than 1,000 bp in length, contained incorrect primer sequences, or contained more than 1 ambiguous base were discarded. Operational taxonomic units (OTUs) were picked using BLAST and the greengenes reference database at a level of 97% similarity [20]. Principal coordinates were generated using un-weighted and weighted UniFrac distances for samples with high and low FP activity (*n*=26) [21,22]. PCoA plots were used to visualize the similarities or dissimilarities between high and low FP activity groups that best represent the pair-wise distances between sample groups. Statistical differences between high and low FP activity groups were tested using analysis of similarity (ANOSIM – available through QIIME) by permutation of group membership with 999 replicates. The test statistic R, which measures the strength of the results, ranges from −1 to 1: R = 1 signifies differences between groups, while R = 0 signifies that the groups are identical. An OTU network-based analysis was used to visualize OTU sharing between sample groups where a bipartite network was generated in which high and low FP activity samples were designated as one node type, and bacterial OTUs were designated as a second node type. A given sample was connected to a given bacterial OTU node through a line (edge) if that OTU was detected in the sample. The number of observed bacterial species and the Shannon index of diversity were compared between high and low FP activity groups following rarefaction of OTUs using a student’s t test. Bacterial taxonomic groups were used for correlation analysis if they appeared in at least 15% of samples. 

### Quantitative real-time PCR (qPCR)

qPCR was performed using the SYBR ® Green PCR master mix (Applied Biosystems, Carlsbad, CA) with primers that amplify the genes encoding 16S rRNA from *Faecalibacterium prausnitzii* (forward, 5'-GATGGCCTCGCGTCCGATTAG-3'; reverse, 5'-CCGAAGACCTTCTTCCTCC-3') and all bacteria (forward, 5'-GTGSTGCAYGGYTGTCGTCA-3'; reverse, 5'- ACGTCRTCCMCACCTTCCTC-3'). qPCR assays were conducted in 96-well plates on an Eppendorf Realplex2 mastercycler thermocycler (Eppendorf, Hauppauge, NY). Each PCR was carried out in a final volume of 25 µl and contained the following: 1 × SYBR green master mix, 0.5 µM of each primer and 10 ng of purified fecal DNA. PCR conditions were as follows: 10 min at 95°C, followed by 40 cycles of 95°C for 15 s, 20 s at 50°C, and 72°C for 1 min. Each plate included duplicate reactions per DNA sample, the appropriate set of standards and a ‘no template’ negative control for each primer set. qPCR standards were generated by PCR amplification of target sequences from genomic DNA of an appropriate positive control strain. Analysis of melting curves confirmed that the fluorescence signal originated from specific PCR products and not from primer-dimers or other artifacts.

### Statistical Analyses

The goal of this study was to correlate the relative (percent) concentrations of specific bacterial groups (identified by 16S rRNA sequence data) with FP activity across the entire study population (*n*=54), whilst adjusting for potential demographic confounders such as age, race, sex, IBS status and body mass index. Age and BMI were treated as continuous predictions. Race, sex, and disease state were dichotomized to non-white/white (1/0), male/female (1/0), IBS/healthy (1/0). FP activity data were transformed to normality using a normal quantile method. We emphasize that we anticipate FP activity to deviate from normality, but such monotonic transformations mitigate the analytical challenges while preserving validity of association results. The abundances of 51 bacterial groups (Phylum=6, Class=9, Order=12, Family=24) identified from V1-3 16S rRNA sequences were correlated with FP activity. 

An unadjusted analysis tested for the association between each bacterial group’s relative concentration and FP activity levels without adjusting for potential demographic confounders, while an adjusted analysis took potential demographic confounders into account. Both adjusted and unadjusted analyses regressed FP activity levels on the concentration for each bacterial group and constructed a wald-test to obtain a *p*-value. For each strategy multiple comparisons were controlled for using a false discovery rate (FDR) at the recommended 10% level [23]. 

For qPCR the percentage of *F. prausnitzii* was determined in all fecal samples ([copies 16S rRNA gene for *F. prausnitzii/*copies of 16S rRNA gene for all bacteria] ×100). The percentage of *F. prausnitzii* was then correlated with FP activity in each stool sample. 

## Results

### Human fecal samples exhibit a wide range of FP activity

**Figure 1:**
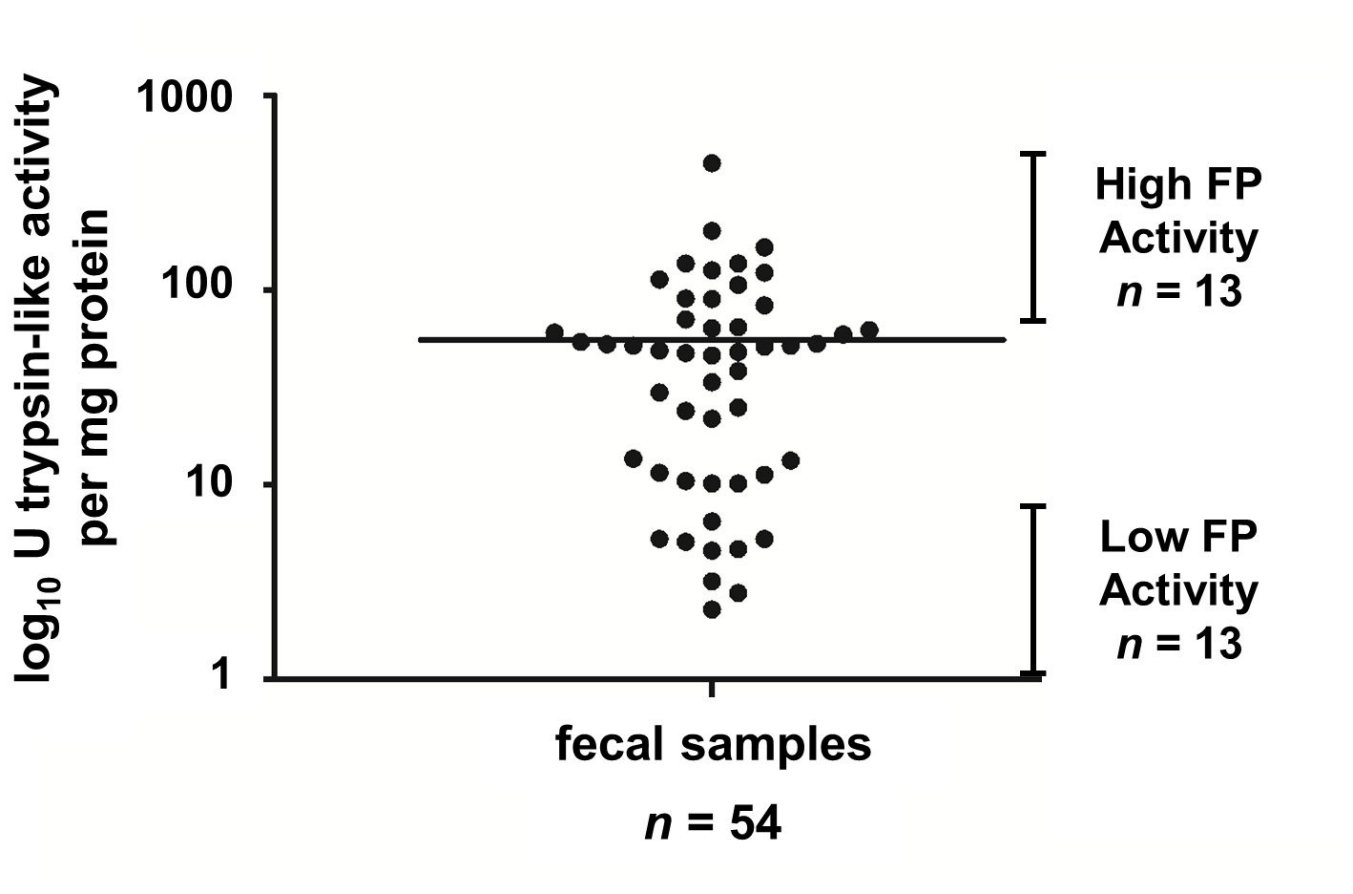
Levels of protease activity in fecal supernatants from all subjects (healthy controls and IBS patients) that participated in this study. Top and bottom quartiles representing samples high and low FP activity, respectively, are indicated.

The source of the fecal samples analyzed in this study included 30 subjects with IBS (23 = diarrhea-predominant, 7 = mixed bowel habit-predominant) and 24 HC, 79% females with a mean age of 35 years. These samples demonstrated a wide range of FP activity (Figure 1). FP activity was ranked from low to high (0-450 U Trypsin/mg protein) and divided into quartiles. The top and bottom quartiles were used to separate and further investigate samples with high (*n* = 13) and low (*n* = 13) FP activity (Figure 1). The FP activity data displayed a strong degree of non-normality. Standard transformations such as *log* and square root did not remove the non-normality. Accordingly, we applied a normal quantile transformation to FP measurements to normalize these data.

### Samples with high FP activity are compositionally distinct to samples with low FP activity

The V1-3 region of the 16S rRNA gene was used to characterize the microbiota in 54 fecal samples. A total of 472,031 16S rRNA sequences with acceptable quality were obtained with an average of 8,741 reads per sample (range: 3,103-16,574; average length = 345bp). Using 3% dissimilarity between sequences to define a ‘species-level’ OTU, we found a total of 1,649 OTUs in the samples analyzed.

**Figure 2:**
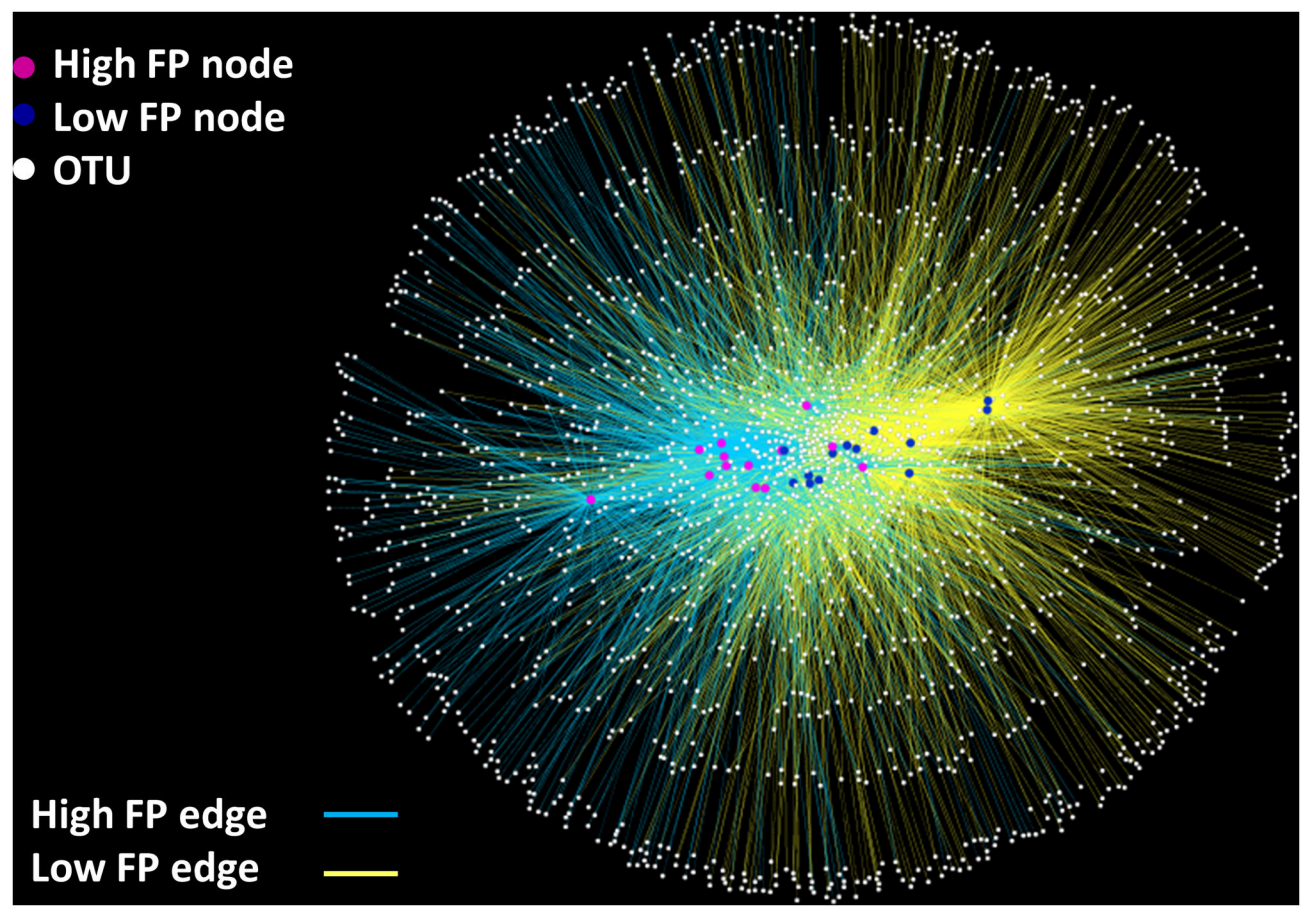
Operational Taxonomic Unit (OTU) network analysis of bacterial communities from samples with high and low fecal protease (FP) activity. Nodes represent high FP activity samples (*n*=13, blue circles), low FP activity samples (*n*=13, yellow circles) samples, and OTUs (white circles). Edges (lines) connecting samples with high FP activity nodes (blue edges) or low FP activity nodes (yellow edges) to OTUs indicate whether a given OTU was found in that sample. The clustering of blue and yellow nodes and edges indicates that samples with high FP activity share numerous OTUs in common, and segregate from the shared OTUs between low FP activity samples.

**Figure 3:**
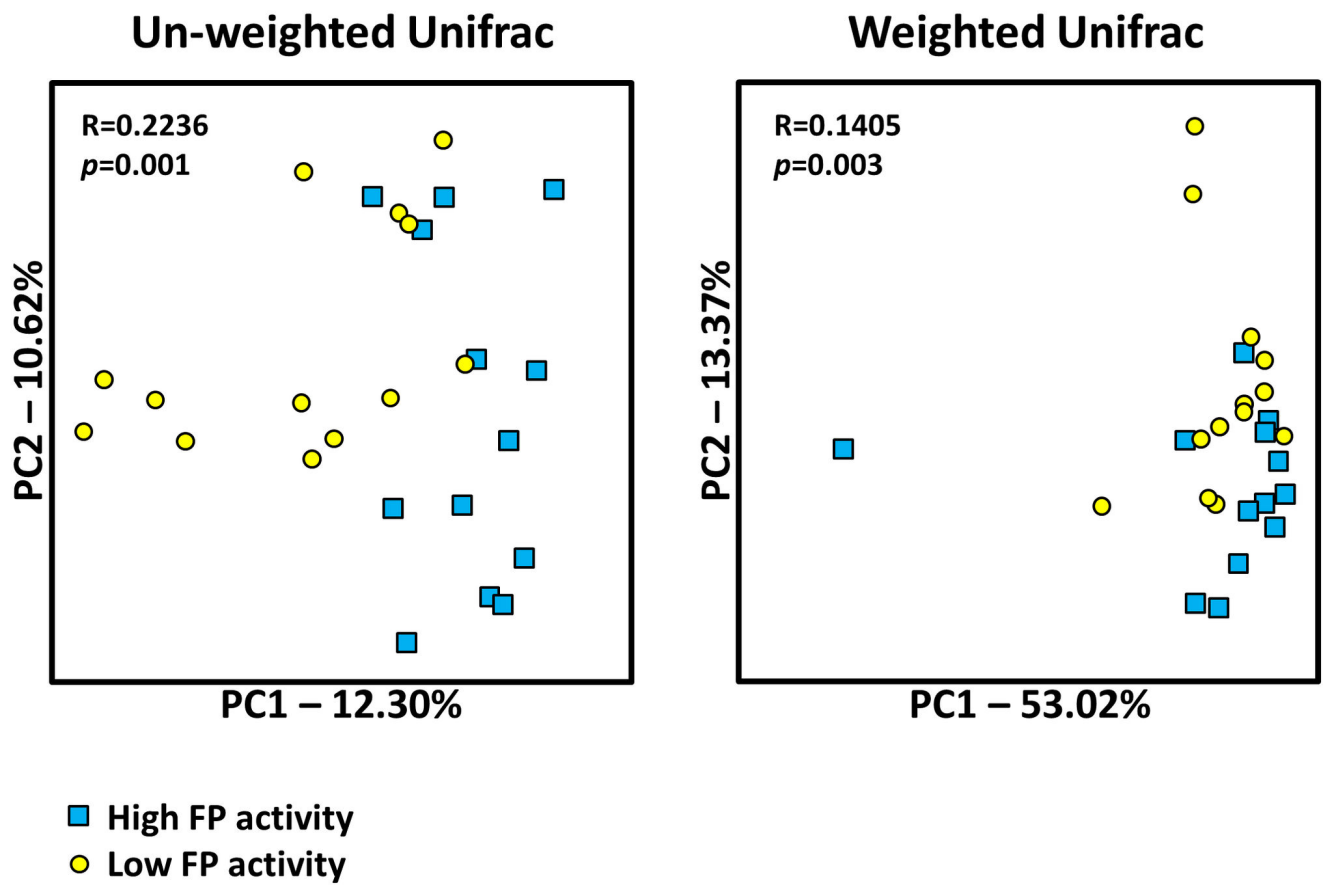
Bacterial community composition analysis between samples with high (*n*=13) and low (*n*=13) FP activity. Principal coordinates analysis (PCoA) plots of un-weighted and weighted UniFrac distances for samples with high (blue circles) and low (yellow squares) FP activity are shown. Analysis of similarity (ANOSIM) demonstrated a significant separation in the composition of fecal microbiotas between high and low FP activity samples using both un-weighted (*p*=0.001) and weighted (*p*=0.003) UniFrac distances. The R statistic (where R=1 and R=0 signifies differences and no differences between groups, respectively) is higher in the un-weighted analysis suggesting the separation between microbiotas is a result of both high and low abundances bacterial species.

Comparison of the composition of the microbiotas between samples with high and low FP activity was first carried out using a bipartite. In Figure 2 high and low FP activity samples are represented by one node type and bacterial OTUs are designated as a second node type. High and low FP activity nodes are connected to OTU nodes via ‘edges’. This OTU network displays a clear separation between high and low FP activity samples. In the second approach Principal Coordinate Analysis (PCoA) of UniFrac distances revealed a significant separation of the microbiotas between samples with high and low FP activity based on un-weighted (*p*=0.001, R=0.22) and weighted (*p*=0.003, R=0.14) distances (Figure 3). 

### Bacterial richness is reduced in samples with high FP activity

**Figure 4:**
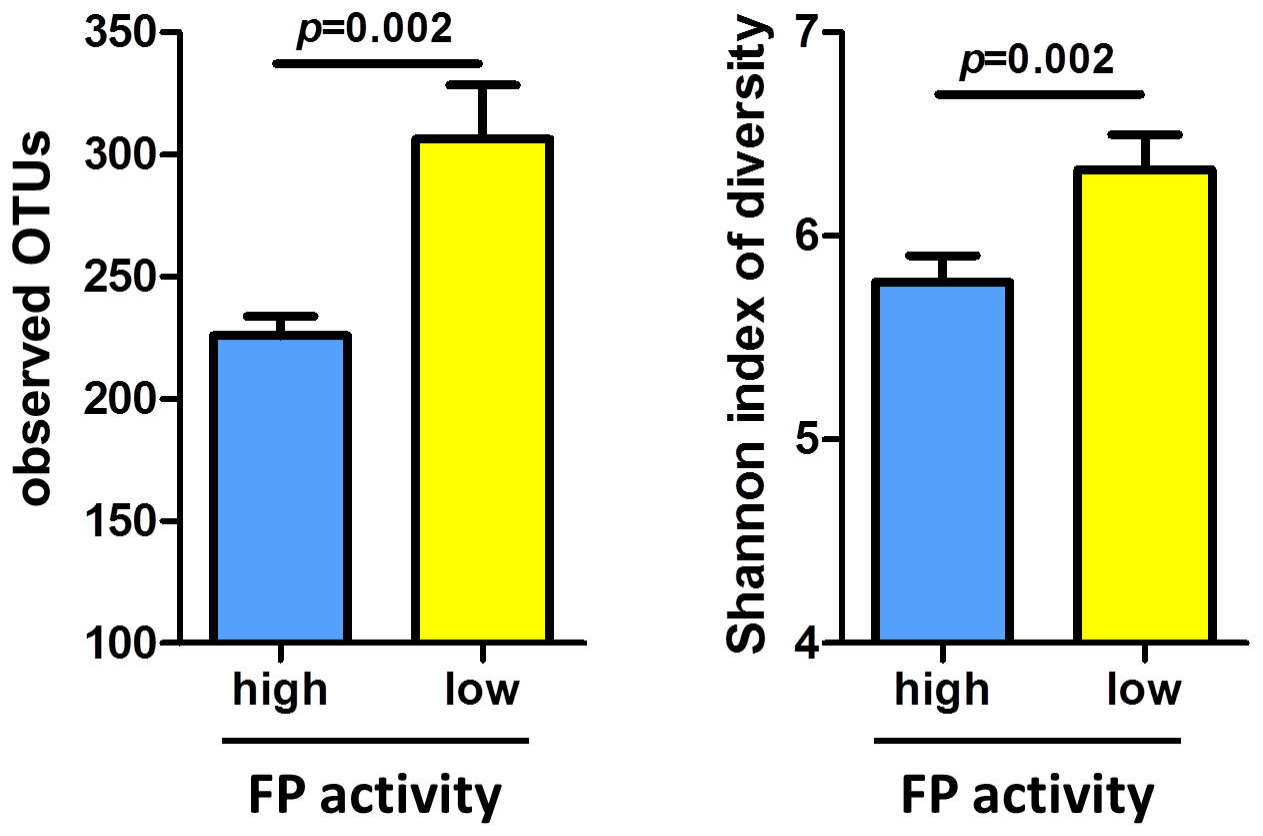
Microbial richness of samples with high (*n*=13, blue) and low (*n*=13, yellow) FP activity. Both the number of observed bacterial species (based on species-level OTUs) and Shannon index of diversity are significantly lower in fecal samples with high compared to low protease activity (*p*=0.002). Error bars represent the standard error.

Bacterial richness was assessed by rarefaction of OTUs found in high and low FP activity samples. A significant decrease in the number of observed OTUs and the Shannon index of diversity was found in samples with high compared to low FP activity (*p* < 0.002) (Figure 4). 

### Specific bacterial taxa are associated with FP activity

**Figure 5:**
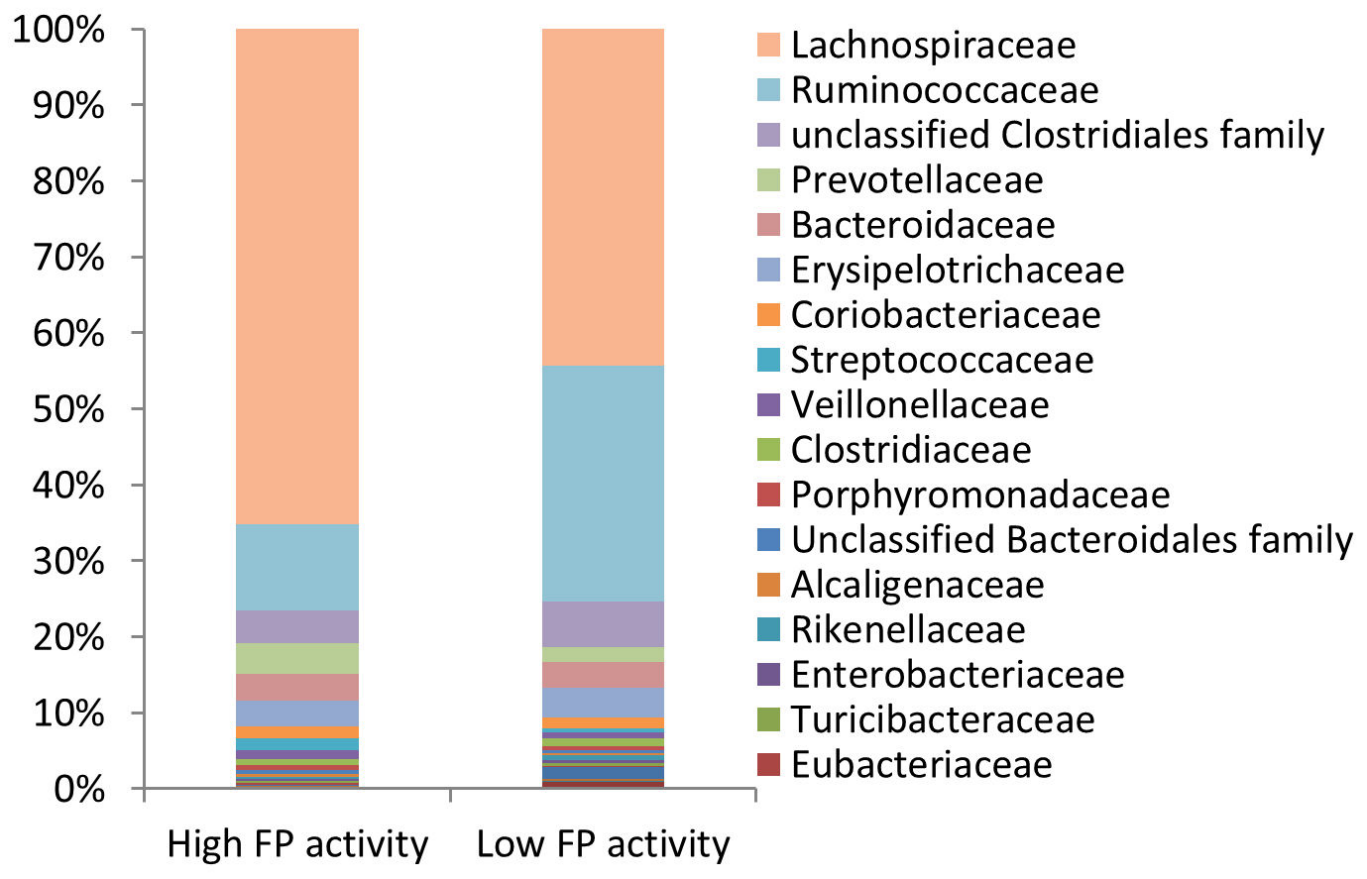
Abundances of Family level taxa in samples with high (*n*=13) and low (*n*=13) FP activity. The composition and abundances of bacterial families differ between the microbiotas of fecal samples exhibiting high and low FP activity.

Family level taxa were found to display compositional differences between samples with high and low FP activity (Figure 5). Correlation of bacterial groups with transformed FP activity levels across the entire study population identified significant associations with specific bacterial taxa at the Family level at an FDR level of 0.1 (Table 1). The *Lactobacillales*, *Lachnospiraceae*, and *Streptococcaceae* groups were positively associated with FP activity across the entire study population, whilst the *Ruminococcaceae* family and an unclassified *Coriobacteriales* family were negatively associated with FP activity (Table 1). 

**Table 1:** Bacterial groups significantly correlated with fecal protease activity.

**Taxonomy**	***p***	FDR*	**Adjusted *p*^¥^**	**Adjusted FDR^†^**	**Correlation coefficient (r)**
*Ruminococcaceae*	0.0003	0.006	0.0011	0.026	-0.46
*Lachnospiraceae*	0.0027	0.032	0.0059	0.071	0.42
*Streptococcaceae*	0.0056	0.045	0.0143	0.086	0.36
*Lactobacillales*	0.0040	0.049	0.0090	0.108	0.37
*Unclassified Coriobacteriales family*	0.0133	0.080	0.0108	0.086	-0.30

^*^False discovery rate (FDR) to correct for multiple comparisons.

^¥^ *p* value adjusted for confounding demographic variables.

^†^ FDR adjusted for confounding demographic variables.

**Figure 6:**
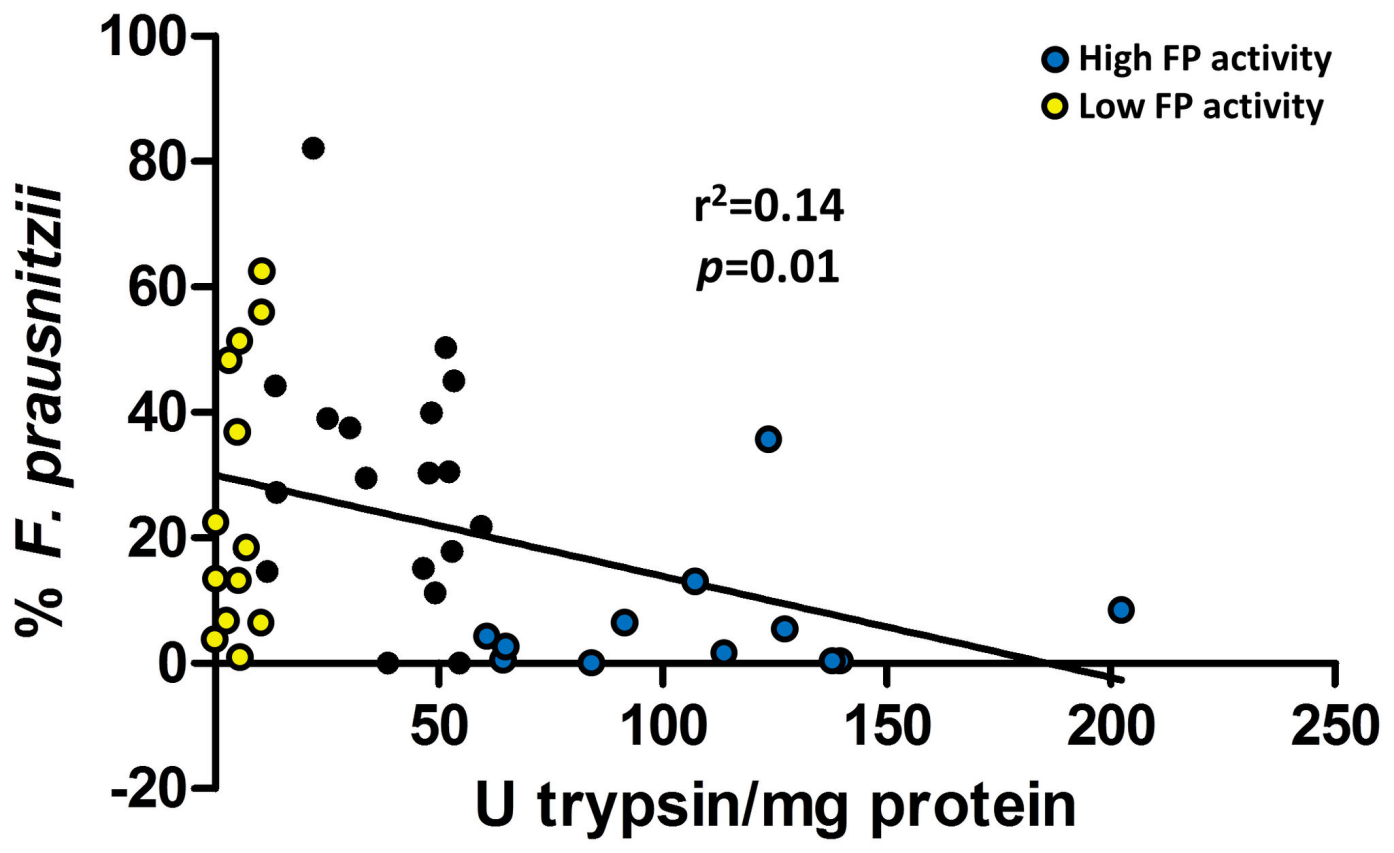
Correlation of *Faecalibacterium prausnitzii* with FP activity. *F. prausnitzii* exhibits a significant (*p*=0.01) negative correlation with FP activity. Blue and yellow circles indicate high and low FP activity samples used in previous analyses, respectively.

The *Ruminococcaceae* family encompasses an important genus that has been reported to be important for GI health (*Faecalibacterium*) [24,25]. *Faecalibacterium prausnitzii* is the only microbial species within this genus. Thus, we determined the abundance of this bacterial species in 53 out of 54 stool samples (one sample repeatedly failed to detect to amplify *F. prausnitzii* sequences) using qPCR. We found a significant (*p*=0.01) negative correlation of *F. prausnitzii* with FP activity (Figure 6). Association of FP activity with baseline demographic data (age, race, BMI, and IBS status) revealed no confounding individual predictors (p=0.67).

## Discussion

Although it has been known for some time that the intestinal microbiota is a significant source of protease activity in the GI tract, to date there has only been one report that correlated specific enteric bacterial taxa with FP activity in the human gut [26]. By analyzing the protease activity of representative enteric bacterial strains and human fecal samples it has previously been suggested that the activity of specific classes of proteases present in human feces are likely to originate from *Bacteroides*, *Streptococcus*, and *Clostridium* species [5]. Interestingly, Midtvedt et al. [26] reported an inverse correlation between *Bacteroides* and fecal tryptic activity, however this association was reported with respect to Crohn’s disease patients and not healthy individuals. Our study used molecular methods to identify the enteric bacterial taxa that are associated with FP activity, thus adding to the knowledge of human protease activity in the human GI tract. 

We found a significant difference in the overall composition of the microbiota between fecal samples with high versus low FP activity. This compositional difference was detected using both un-weighted and weighted UniFrac distances. The former calculates phylogenetic distances between samples based on the presence or absence of an OTU, whilst the latter calculates distances based on the presence and abundance of OTUs [21,22]. A significant difference between the microbiotas of high versus low FP activity samples using both un-weighted and weighted analyses suggests that the difference in microbial communities between these groups is based on both dominant and low abundance bacterial taxa present in fecal samples. This finding is supported by the estimation of α-diversity measures (rarefaction) between fecal samples with high versus low FP activity. We found that the number of observed bacterial species (based on species-level OTUs) and Shannon index of diversity were significantly lower in fecal samples with high FP activity. The biological relevance of a diverse intestinal microbiota has not yet been established; however it is important to note that the diversity of microbial species in the gut is reduced in patients with intestinal diseases such as IBD and IBS [15,27]. Together, these findings indicate that individuals with high FP activity have lower numbers and diversity of bacterial species in their intestine and harbor a microbiota that is distinct from individuals with low FP activity. 

When we applied a stringent association analysis and adjusted for confounding demographic data across our entire study population between FP activity and specific bacterial taxa, we found the *Lactobacillales*, *Lachnospiraceae*, and *Streptococcaceae* groups and an unclassified *Coriobacteriales* family were positively associated with FP activity. The *Lactobacillales* order encompasses seven bacterial families [20] including the *Streptococcaceae* family. The *Streptococcaceae* family is likely the driving force behind the association between the *Lactobacillales* order and FP activity. *Streptococcaceae* encompasses the *Lactococcus*, and *Streptococcus* genera, which are both lactic acid producers. Although, this family contains a probiotic organism (*Lactococcus lactis*) it is dominated by *Streptococcus* species: one of the groups postulated to be associated with protease activity in the intestine [5]. Interestingly, members of the *Streptococcus* genus have been found to produce proteases with a broad range of functions [28-33]. 

In all the samples studied we found that the *Lachnospiraceae* were the dominant bacterial group at the family level (on average approximately 50% of 16S rRNA sequences). This family encompasses twenty one bacterial genera with diverse functions [20] and contain *Clostridium* species. It has previously been shown that the abundance of *Clostridium* species coincide with proteolytic activity in human fecal samples [34], which is in line with our findings. Interestingly, specific members of the *Lachnospiraceae* family (*Ruminococcus torques*, *Ruminococcus gnavus*, and *Clostridium coccoides*), have been reported to be associated with IBS and IBD [24,27,35-37]. 

We also found that the *Ruminococcaceae* family was negatively correlated with FP activity. The *Ruminococcaceae* are a family of obligate anaerobes that encompass twelve bacterial genera, some of which are responsible for producing short-chain fatty acids in the gut (including: *Butyricicoccus*, *Clostridium*, *Faecalibacterium*, *Ruminococcus*, *Subdolingranulum* spp.). *Faecalibacterium prausnitzii* is the only member of the *Faecalibacterium* genus, and this bacterium is believed to be protective of inflammation in the gut [24,25]. Indeed, our qPCR analysis confirmed an association between *F. prausnitzii* and FP activity. The *Butyricicoccus* genus contains *Butyricicoccus pullicaecorum*, a butyrate producing microbe [38]. Butyrate plays an important role in the metabolism and normal development of colonic epithelial cells, and has been implicated in protection against cancer and ulcerative colitis [39]. Additionally, we found that an unclassified *Coriobacteriales* family was associated with FP activity. Given the lack of information regarding this group of bacteria, we cannot speculate on the relevance of its relationship to FP activity. As the *Ruminococcaceae* family encompasses beneficial bacterial species and the *Streptococcaceae* family encompasses protease producers, it is tempting to speculate that a microbiota associated with low FP activity is associated with a more beneficial to the host than a microbiota with high FP activity. 

Unlike previous studies that observed a significant increase in FP activity in IBS patients compared to healthy controls [6,10], we did not find a substantial difference in FP activity between these groups in the current study. However, the goal of our study was not designed to compare protease activity between IBS patients and healthy individuals, but to investigate the association between intestinal protease activity and specific bacterial groups in the gut microbiota. Indeed, we have previously reported differences in enteric microbial communities between IBS patients and healthy individuals [15]. 

In conclusion, we report a distinction between the microbiotas within fecal samples with high and low FP activity. The distinction between these microbiotas is likely due to the presence of low microbial diversity and the absence of bacterial taxa in fecal samples with high FP activity. Our results provide evidence of an association between fecal protease activity and specific members of the intestinal microbiota. One of the strengths of our study includes the diverse source of fecal samples (i.e., from HC and certain types of IBS patients) that enable the analysis of human specimens with a broad range of FP activity. However, although our data demonstrate a clear association it does not determine whether these bacterial groups are a cause for the increase in protease activity or a response to the change in the intestinal luminal environment. FPs have been shown to have the potential to alter intestinal physiological functions (e.g., motility, sensation, permeability, immune function) [10,14,40,41] which may by themselves be associated with alterations in the intestinal microbiota. Furthermore, the relative contribution of the altered microbiota in individuals with elevated FP activity could be responsible for the alterations in GI physiology and the pathogenesis of certain GI diseases e.g., IBS and IBD [11,42-44]. Our study findings provide a basis for further research, including metagenomic sequencing, to investigate the role of the bacterial groups identified in our study, and the proteases they produce, with the pathogenesis of GI diseases.
